# Impact of Dining Hall Structural Changes on Food Choices: A Pre-Post Observational Study

**DOI:** 10.3390/ijerph17030913

**Published:** 2020-02-01

**Authors:** Julia Carins, Sharyn Rundle-Thiele, Rimante Ronto

**Affiliations:** 1 Social Marketing at Griffith, Griffith Business School, Griffith University, Nathan, QLD 4111, Australia; s.rundle-thiele@griffith.edu.au; 2 Land Division, Defence Science & Technology Group, Scottsdale, TAS 7260, Australia; 3 Department of Health Systems and Populations, Faculty of Medicine and Health Sciences, Macquarie University, NSW 2109, Australia; rimante.ronto@mq.edu.au

**Keywords:** food choice, observation, food environment, structural change

## Abstract

Change that benefits individuals and organisations while delivering health outcomes and benefits society requires a research focus that extends ‘beyond the individual’ to environment shapers. A pre-post observational study assessed two food provision structural changes to understand the role food service environments had on food selections. Diners were observed prior to (lunch *n* = 1294; dinner *n* = 787) and following (lunch *n* = 1230; dinner *n* = 843) structural changes in a buffet-style dining room—including provision of a healthy convenient meal alternative for lunch (healthy lunch bag), and a pleasurable dinner (make-your-own pizza). Food choices shifted with 19% of diners opting for a healthy lunch bag and 29% of diners selecting a pizza dinner, moving away from the existing buffet. Examination of selections by those continuing to select from the concurrent buffet selections established that the availability of healthy alternatives in the buffet partially explained food choices, moderating any observed changes in food selections. The food service sector is a promising avenue through which dietary behaviours can be improved. Further studies, particularly those that measure selections over the longer term, and that include measures of satisfaction and profit, are needed.

## 1. Introduction

Complex health and social issues such as unhealthy dietary behaviours are characterised by multiple layers of influence, therefore, investigation and strategic action needs to be directed wider than individuals at the centre of the issue. Social marketing, and other behavioural change sciences, have predominantly focused on fostering individual change [1], however, broader conceptualisations are now focusing on identifying factors that perpetuate detrimental behaviours [2], and/or impede beneficial behaviours [3]. Policy and regulation are ‘beyond-individual’ approaches [4,5], and modification of the food service sector is an alternate path of action [3].

In recent decades, dietary behaviours have dramatically worsened in many countries [6,7]. Unhealthy dietary intake is a major risk factor contributing to overweight and obesity, and poor diet is a leading cause of mortality and disability worldwide [8,9]. A healthy diet may prevent the most severe outcomes, however, they also play an important role in recovery from illness or injury; normal physical, mental and emotional functioning; and in peak productivity or performance [10,11]. The need to increase healthy food consumption is clear. Individual conceptualisations of unhealthy dietary behaviour and obesity are now considered too simplistic [12]. Individuals exert some control over their food choices, however, dietary behaviours are highly complex and are also influenced by surrounding factors including those in the food environment [13]. 

The food service sector can play an integral role in influencing consumer dietary behaviours. Consider the institutional food sector—workplaces, universities, schools, colleges, military settings, nursing homes or prisons—which provides between 10%–15% of all foodservice meals [14]. Institutional food settings often comprise a buffet, which may lead to excessive food intake [15]. For example, studies have found eating in all-you-can-eat dining halls accounted for 20% of the variance in weight gain in university students [16], and on-campus students gained more weight than students commuting from home [17]. Alterations to food services have been identified as one method within a wider set of actions to combat obesity [12,18,19]. Yet, a review of food service initiatives indicates that individually-focussed awareness raising schemes remain the most common strategies used by food service providers [20], despite being ineffective or delivering modest changes to consumer dietary behaviours at best [21]. Few studies report employing strategies that are structural in nature [22,23], and the evaluation of structural interventions is challenging [24]. Marketing is underpinned by a philosophy focussed on voluntary exchange relationships that deliver value for both customers and stakeholders [25]. This emphasises the need to offer people choices that they value, that also deliver outcomes sought by organisations and by extension, society as another stakeholder in the process. Important criticisms (such as preaching to consumers) have been leveraged at policy, public health and other behaviour change approaches that are involuntary [26,27]. A shift in behavioural change efforts away from individuals, to the factors that food services can control, ensures that one group (e.g., consumers) are not unfairly stigmatised and blamed for inappropriate food choices. By co-creating solutions and building healthy food alternatives that people voluntarily choose and are willing to pay for, changes can be achieved that benefit both the individual consumer, and society more generally.

This study addressed these gaps evaluating a structural change allowing freedom of choice that did not seek to raise awareness, inform nor engage individuals in healthy eating appeals. The aims of this study were twofold. First, this study provided a case study outlining two structural changes, each of which provided alternative food choices for consumers—nutritionally balanced and less healthy. Second, this study assessed consumer food choices, using observational methods, to determine whether uptake of the new options would substantially alter the healthfulness of the choices made by the population within the dining environment, outlining a method that can be used to assess the degree that structural changes increase (or not) healthy eating. 

## 2. Materials and Methods 

This study was performed in an on-campus dining facility. Participants were young adults undertaking undergraduate education/vocational training and living on campus, with a higher proportion of male attendees (approx. 75% male 25% female as obtained from published intake records for 2017/2018). Although the campus was city based, it was located ten minutes away, by car, from alternative shopping/commercial areas, therefore the dining facility represented a close and convenient place to obtain a meal. Opening times spanned two and a half hours for lunch (two provide for different class time breaks) and two hours for dinner. Diners paid a set meal price on entry, which generated a paper ticket. This ticket was dropped into a container at the beginning of the mains counter, visually indicating to the caterers that the diner had paid for the meal.

### 2.1. The Structural Changes

Initially, food provision was in the form of a buffet-style meal. Diners chose from food counters (or bains-marie), containing a main dish (diners made one choice from five to six choices offered); and side dish options from counters containing hot vegetables, salads and sandwich ingredients (e.g., breads, sliced cheese, cold cuts, sliced tomato, sliced cucumber, grated carrot and similar items). Diners could visit any or all of the hot vegetable, salad and sandwich ingredient counters, and multiple choices were allowed from these counters. For example, a diner might select one main, visit the vegetable counter and take two vegetables, move to the salad counter to add some salad, and then move to the sandwich fillings counter to make their last selection (perhaps some slices of tomato). A sign was placed on the mains counter indicating diners should only take one main, and a caterer was usually present in this area, which may have encouraged compliance with the sign. It would have been possible to return for a second pass at this counter, if the diner did not make it obvious to the caterer that they had not placed a paper ticket in the container.

As part of a servicescape makeover (new furniture, plants, and decorative screens to break up a large dining room) two new meal options were provided—a nutritionally balanced meal at lunch (a paper bag lunch); and a non-nutritionally balanced meal option at dinner (make-your-own (MYO) pizza). The paper bag lunch was designed by a nutritionist to provide one third of the daily calories/kilojoules for an adult, with a nutritionally balanced spread of macronutrients, and was therefore considered a ‘nutritionally-balanced’ option. From the designated counter, diners filled a paper bag with four items: (1) a wrap, sandwich or boxed salad (2) a small snack (3) a piece of fruit (4) a bottle of water. Catering staff were present to assist and to ensure only four items were selected. The MYO pizza (by its very nature) was composed freely by diners, using a pre-made base and prepared toppings. Even lightly topped, or vegetarian pizzas contain sodium or saturated fat above recommended levels [28,29], therefore, all pizzas were considered ‘non-nutritionally balanced’. The new options were designed to appeal to consumers preferences for those meals, that is, lighter convenient meals at lunch, and heavier pleasurable meals at dinner [30,31]. These new options were offered as a complete meal and were an alternative to the buffet-style meal—which remained available for diners to choose should they not wish to select one of the new options. Buffet-style meal provision continued in line with the cyclical menu being used prior to the structural change and did not radically change (beyond variations in number and types of dishes, which was monitored throughout the study). Structural changes occurred without promotion, and without being priced differently (a set meal price was paid on entry). To summarise, the new options were structural changes to availability made within a real-world food choice environment. 

### 2.2. Study Methods

The study employed a pre-post evaluation research design, triangulating two observational data collection methods, to quantify and describe behavioural patterns that occurred before and after two structural environmental changes. Observational methods alleviate participant burden, and remove recall and social desirability biases intrinsic to self-report methods [32]. Method 1, direct observation, recorded the number of diners who selected an option from each food counter, capturing all choices made by the total dining population during mealtime, eliminating potential biases related to self-selection within a data collection. Method 2, plate photography, captured individual food choices for a subset of diners following the methods of Carins et al. [28]. Briefly, this involved a researcher/photographer approaching diners spontaneously at the point where they had finished their food selection, announcing themselves as a researcher collecting information about the food customers selected in the dining hall, and asking whether a photograph could be taken of their meal. Photographs were taken to capture as much of the food as possible (usually directly from above). Less than a handful of diners refused plate photography, however, it was still relatively easy for diners to avoid plate photograph by taking a route to a table that did not involve passing close by the photographer. Participants were aware the research was investigating food choices, but they were not fully informed of the exact study purpose (food choice changes), to minimise social desirability bias and/or the observer effect where participants modify aspects of their behaviour in response to awareness of being observed as far as possible [33]. 

Ethical approval was obtained for the research (GU 2017-023), which involved observing behaviour in a public place (food counter usage) with consent for plate photography provided by participating diners. Four meals were observed at each time point (two lunches and two dinners). Diners used swipe cards to enter the dining room and pay for the meal, which provided an accurate record of the number of diners attending each meal.

### 2.3. Data Analysis

Direct observation data captured the uptake of the new options, and the counts of selections from each counter, and were compiled and compared with attendance data. Chi-square tests were conducted to determine differences in the count data before and after the structural change. This observation data was used to examine the uptake of the new options—one was nutritionally balanced (healthful choice) the other was non-nutritionally balanced (non-healthful choice). Observation data was also used to examine whether the introduction of new options resulted in marked decreases in the use of any of the buffet counters. 

The direct observation results did not show if food selections changed at an individual level, beyond those who migrated to the new options. Therefore, plate photography was used with a subset of the dining population to examine individual selections, focussed on meals selected from the buffet (from the bains-marie). Plate photographs were examined, and food selections on each plate were matched to the list of food choices (dishes) available for that meal in the buffet sourced from the menu. Furthermore, the aggregate results did not indicate the healthfulness of food selections (beyond the healthful paper lunch and the non-healthful pizza choices). For example, in the buffet hot vegetable section there may have been steamed broccoli alongside cheesy potato bake. Therefore, a classification process was used to determine the healthfulness of selections on each plate of food. As a separate and independent process, each available food dish from the menu was classified as red (least healthful—such as cheesy potato bake), orange (moderately healthful) or green (most healthful—such as steamed broccoli) using a healthfulness scheme [28]. An overview of the classification is provided in Appendix A. The results of the photo examination (counts of selections from available dishes) and the classification of food dishes (healthfulness categorisation of each available dish) were then combined so that each plate of food could be expressed as the number of red (least healthful), orange (moderately healthful) and green (most healthful) choices. This allowed for a comparison of the healthfulness of selections before and after the two food provision changes. The number and type of dishes available on the menu (provision) varied meal to meal and was captured by the research team. 

Independent samples t-tests were used to compare choices of red, orange and green selections (by diners choosing from the buffet) before and after the structural change. These tests did not take into account the variation in provision, so analysis of covariance was used, using provision of orange and green options as covariates (provision of red options was considered stable). Visual inspections of histograms, p–p plots and q–q plots indicated that the data could be considered reasonably normally distributed; however, results were cross-validated with non-parametric equivalents (specifically Mann–Whitney U tests, and Quade’s nonparametric analysis of covariance) to ensure that any degree of non-normality did not distort the findings. 

## 3. Results

### 3.1. Observation

**Figure 1:**
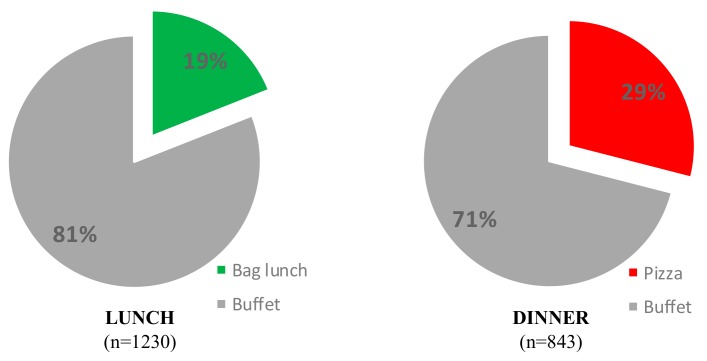
Uptake of new meal options measured via direct observation.

Four consecutive meals (two lunches and two dinners) were observed both before and after the changes. Attendances were similar before (lunch *n* = 1294 (lunch 1 *n* = 646, lunch 2 *n* = 648); dinner *n* = 787 (dinner 1 *n* = 389, dinner 2 *n* = 400)) and after the structural change (lunch *n* = 1230 (lunch 1 *n* = 583, lunch 2 *n* = 647); dinner *n* = 843 (dinner 1 *n* = 431, dinner 2 *n* = 412)). Offerings at lunch and dinner were broadly similar (refer to Appendix A), similar proportions of main, hot sides, salads and sandwich ingredients were offered. The exception was the absence of a separate sandwich fillings counter during dinner after implementation—although some sandwich fillings were provided in the salad bar at this time. Uptake of the new options (healthy paper bag lunch; MYO pizza dinner) was substantial in each case. For the lunch meal, the paper bag meal was selected by 19% of diners, and the buffet was utilised by the remaining 81% of diners who did not choose the new option. At the dinner meal, the MYO pizza meal was selected by 29% of diners. The remaining 71% of diners utilised the buffet (see Figure 1).

As both new options represented a complete meal offering it was expected selections from the buffet food counters would be different (lower) after the structural change. At lunch, observation data indicated that fewer diners selected from the mains counter (χ^2^ = 36.932, *p* < 0.001) and the sandwich fillings counter (χ^2^ = 394.334, *p* < 0.001) whereas the change was minimal in terms of number of diners selecting from the hot vegetable counter (χ^2^ = 5.919, *p* = 0.015) and salad counters (χ^2^ = 0.024, *p* = 0.876). This data is shown in Table 1. 

It must be emphasised that this observation data indicates patronage at each counter by the entire dining population for each meal and does not indicate amounts selected by each individual diner. Therefore, a reduction in patronage at a counter suggests ‘movement’ away from those offerings. This aggregate pattern suggests that those who adopted the new lunchtime option (paper bag lunch) had most likely selected from the counter with sandwich fillings before the changes, either for all choices on their plate, or together with a choice from the main counter. 

For dinner, observation results show decreases in the number of patrons selecting from each of the counters to a similar degree (refer to Table 1), for main dishes (χ^2^ = 168.69, *p* < 0.001), hot vegetables (χ^2^ = 136.416, *p* < 0.001) and salads (χ^2^ = 19.869, *p* < 0.001). Note that a separate counter with sandwich fillings was not provided during the dinner meal after implementation, however, it was noted that the caterers provided some sandwich fillings in the salad bar at dinner at this time. Given this pattern shows a reasonably similar reduction across all counters, reflective of the fewer numbers utilising the buffet, it suggests that those who migrated to the pizza option could be considered to be a uniform subset of the larger group. 

It is important to note here that the sum of the new options (a complete meal) and the main dishes (where diners were only supposed to choose one main) exceeded 100% of diner numbers after the structural change. This occurred at both meals (19% chose a paper bag lunch plus 85% a lunch main meal which equals 104%; 29% chose a MYO pizza plus 77% a dinner main meal which equals 106%). This could indicate some diners (4% at lunch or 6% at dinner) returned to select a second main dish. This practice may have also occurred prior to the changes but was not obvious in our data due to use of the sandwich bar by those making sandwiches only (and not taking a main) and by those adding sandwich ingredients as side dishes. Expressed another way, at lunch there may have been >7% choosing to make a sandwich only (and not taking a main) plus 93% choosing a lunch main meal which equals >100%. This is discussed further in the limitations.

### 3.2. Plate Photography

Once uptake of new options had been quantified, plate photography was used to examine patterns in food selections by diners who had composed a meal from the buffet. This involved a subset of the total dining population at both timepoints. Prior to the structural change, 299 plate photographs were taken, and 351 were taken after the change. Four photographs were excluded as unsuitable for analysis (*n* = 3 food obscured; *n* = 1 items partly eaten). Before the changes, this data capture represented 12% of lunch diners (159 of 1294) and 18% of dinner attendees (139 of 787). After changes, 73 photographs containing new options were held aside (2 paper bag lunch and 71 pizza). The new options were complete meal offerings, and as such were not able to be analysed in the same way as the buffet plate photographs. For example, the nutritionally balanced paper bag lunch could have been considered a ‘green’ selection, and the MYO pizza would have been classified as a ‘red’ selection, but each would have been counted as one selection, in contrast to buffet plates which contained, on average, five selections (one main and four sides). Removal of these photographs resulted in a dataset representing 22% of lunch buffet users (224 of 1000), but only 9% of dinner buffet users (51 of 599). 

Analysis showed the total selections on each plate was similar for each meal, and across timepoints—diners typically chose five selections, a main dish, and four sides (any combination of vegetables, salad or sandwich fillings). In terms of healthfulness, the plates typically contained zero or one red selection, one or two orange selections, and two to four green selections. Mean differences in red, orange and green buffet selections, per plate, before and after the structural change are shown in Table 2; parametric test results were all confirmed with non-parametric tests. 

At lunch, there were higher mean numbers of red and orange selections afterwards compared to before, along with a lower mean number of green selections. These differences were small but significant, for example, a mean difference of 0.21 red selections was equivalent to a 4% increase in the proportion of red selections on a plate (0.65 red/5.56 total = 12% before vs. 0.86 red/5.27 total = 16% after). Similarly, a decrease of 0.90 green selections was equivalent to a 14% reduction in the proportion of green selections on a plate (3.87 green/5.56 total = 70% before vs. 2.97 red/5.27 total = 56% after). In summary, although 19% of lunch diners were assured of having a healthful meal (by taking the new option) after the structural changes, the remaining 81% of buffet users were eating slightly less healthfully compared to buffet users before the changes. 

At dinner, there were a higher mean number of red selections, along with lower mean numbers of orange and green selections. Drawing the example, the mean difference of 0.91 red selections was equivalent to an 18% increase in the proportion of red selections on a plate (0.40 red/5.50 total = 7% before vs. 1.31 red/5.16 total = 25% after). Similarly, a decrease of 0.76 green selections was equivalent to a 10% reduction in the proportion of green selections on a plate (3.94 green/5.50 total = 72% before vs. 3.18 red/5.16 total = 62% after). In summary, 29% of diners were having a less healthful meal (by taking the new option) after the structural changes, and the remaining 71% of buffet users were eating slightly less healthfully compared to buffet users before the changes. 

However, these tests did not take into account the variation in alternatives available to diners—fluctuations in provision will constrain or permit choices from each category. The number of buffet options offered (provision) was captured at each meal to understand the extent that healthful food alternatives were available for selection from the buffet, and to capture the degree of variability in buffet offerings (see Table 3).

The buffet options showed a relatively consistent pattern of availability. For example, at lunchtime, 16% of the options were categorised as least healthful, 26% moderately healthful and 58% most healthful prior to the changes. These proportions differed by one percentage point (or less) at lunch after the changes, and the total number of options was similar. However, at dinner, fewer of the moderately healthful and most healthful options were available after the structural change compared to before, which resulted in a lower total number of available options provided at dinner after the change. This reduction was due to the absence of the counter containing sandwich fillings—or more specifically, the concurrent reduction of some salads, and inclusion of some sandwich fillings in the salad bar—reducing the overall number of green dishes. Proportionally, this equated to a higher proportion of red options, and lower proportion of orange options available in the buffet at dinner after the changes, suggesting that this variation in provision could have contributed to the greater number of red selections, and fewer orange and green selections, after the changes.

Given the large variation in the number of orange and green options provided at dinner, the influence of this varying provision was examined. An analysis of covariance found the provision of orange and green options were significant covariates of food selection (provision of red options was considered stable). The provision of orange and green options explained 31% of the variance in red selections (R^2^ = 0.305, F (1186) = 27.277, *p* < 0.001), and 14% of variance in orange selections (R^2^ = 0.139, F (1186) = 10.050, *p* < 0.001), but only 4% of variance in green selections (R^2^ = 0.044, F (1186) = 2.822, *p* < 0.04). All analysis of covariance findings were confirmed by the Quade’s nonparametric analysis of covariance test. 

## 4. Discussion

In this study, structural changes were made to food provision within a buffet-style dining environment, including the introduction of new meal options (a nutritionally balanced lunch meal, and a non-nutritionally balanced dinner meal). It is important to note that these structural changes did not have an explicit aim of increasing healthy food selections overall, however one structural change (the healthy lunch bag) did offer a nutritionally balanced lunch alternative. This demonstrates that the food service industry can play a positive role in the prevention of obesity and chronic disease, by introducing valued healthy alternatives via structural environmental changes, but without any concurrent information provision. Few studies report employing strategies of a structural nature to improve consumers’ food choices [20,21,23,34]. Examinations of food service initiatives and policy interventions demonstrate that individually-focussed change efforts such as awareness raising dominate [20,35]. Although the findings of this study should be treated as preliminary, the findings show promise, offering empirical data demonstrating that consumers can be stimulated to change food choices without an individually targeted appeal. For example, in this study 19% of lunch diners, and 29% of evening meal diners opted for the alternatives introduced. Further studies are now required to confirm and more broadly examine the impact of such structural changes. The percentage of diners selecting the new options were at similar levels nine months after this research study, suggesting diners considered these options valuable rather than simply novel (new options maintained 20% to 30% usage; data sourced from meal by meal monthly production records, with data provided by the Business Innovation Manager; Catering Company). Although we did not conduct follow up data collection using the same methods to verify how close ongoing usage was to our initial findings, these records suggest a similar pattern continued. This is notable, as maintenance of new dietary behaviours is an important and challenging issue.

Calls for behaviour change efforts to be extended beyond individuals, towards the environments that shape and determine behaviours, have existed for some time [12,36]. Research and practice that address poor dietary choices is dominated by interventions that focus on the individual whose behaviour needs to change (for example, see [37,38]). Calls for social marketing to extend research and practice beyond individually-focussed efforts towards environmental influences have long been evident [4,36], yet a consumer myopia continues to dominate [26]. The wider health field has articulated the need to change environments to better support healthy behaviour [23,39,40]. This study indicates that the food service sector can play an integral role in stimulating positive behaviour change through structural changes to food environments. Consider the rate of uptake of a new healthy alternative in this study (19% of the population) compared to rates of behaviour change in typical diet/exercise communication campaigns (4–8% of the population) [41,42,43]. Equally, the results of this study highlight the role that food services can have in creating less healthy outcomes given 29% of diners were encouraged to switch to a non-nutritionally balanced meal option. To positively change dietary behaviours, there needs to be an increased focus on presenting healthier options conveniently or reformulating appealing foods to ensure that they are healthier. Food service providers also need to be aware of the impact that available alternatives have on food choice. In this study, provisioning in the buffet varied between study time points with fewer healthy options available after the structural change, thereby limiting the opportunity for diners to make healthful food selections. Analysis of individual food choices (without accounting for availability in the buffet) suggested the increased selection of less healthy foods by buffet diners. However, when the availability of alternatives was taken into account, the degree of change was moderated, with availability explaining as little as 4% and as much as 31% of food choice. 

Evaluations of structural changes prove challenging, and rarely measure consumer choice outcomes, instead focusing on the degree to which structural changes were implemented (assuming faithful implementation results in improved consumer health), or relying on self-reports and less accurate means to assess behavioural change [20,21,35]. Responding to these gaps in the literature, this study assessed a structural change, which involved the addition of convenient meal options at lunch and dinner. The potential impact of changes was evaluated using an observational plate photography and food classification method [28] to determine the extent of change in food choices. 

Food service retailers can play an important role in obesity and disease reduction, and in facilitation of a diet that supports physical, mental and emotional wellbeing, by changing the range of alternatives offered to individuals for selection [44]. However, motivating these changes is challenging—food providers hold concerns of low customer demand for healthier alternatives, and a fear of potential revenue losses [45]. This study indicates healthy options, or bundles of healthy options can be formulated and offered to consumers to prompt healthy choices. Taste is considered the foremost influence on food choice, but other factors (cost, nutritional value, and convenience) are also highly influential [46]. Therefore, products that provide benefits in addition to health, such as convenience and taste (for example, the healthy lunch bag), can be used to stimulate food choice. In other studies, concept testing has shown that revenue and customer satisfaction can be maintained when healthy alternatives are offered, even in settings that offer predominantly unhealthy options [47]. Therefore, financial benefits, rather than revenue losses may accrue to retailers who can source and provide cost-effective healthy alternatives. 

Although preliminary, the findings from this study show the impact that the food environment can have on food choices, which is supported by findings from other studies [48,49,50]. Voluntary or mandated schemes that focus on increasing the availability of healthy alternatives, for consumers to freely choose, offer a more cost-effective means to combat unhealthy dietary practices compared to clinical care models, which have been criticised as expensive and unsustainable [51,52]. Furthermore, a high number of the ‘most healthful’ alternatives (~60%) were available in this buffet setting, suggesting a high benchmark can be set for the provision of healthful alternatives. Several Australian state governments have set benchmarks including a minimum of 50% [53] or a specified 75% or 80% of healthy alternatives within their facilities [54,55]. Food environment audit measures can be used to monitor both provision (c.f. [56,57]), alongside measures of the consumer response within the food service sector to track progress, and monitor the sector’s commitment to delivery of healthful food alternatives. 

### Limitations and Future Research Directions

This study must be viewed in light of its strengths and limitations, many of which represent opportunities for future research. Firstly, this evaluation was cross-sectional in nature, and even though individuals may not attend every meal or every day, longitudinal studies are needed to determine the impact on individuals who may dine repeatedly in this environment. These studies should capture the age and gender of diners so that the examination of any group differences in food selection can be investigated—this is especially important given women are often more motivated and knowledgeable with regard to nutrition [58,59]. The findings from this study should be considered as preliminary evidence requiring confirmation, and extension, employing a larger suite of evaluative measures. As a starting point, this could include the inclusion of both healthful and non-healthful novel alternatives at the same time. Then, the impact of healthful alternatives that require some effort (healthful MYO alternatives) could be tested. Further MYO options were planned for this environment—including nutritionally balanced MYO alternatives. Observations of the uptake of other MYO alternatives, including nutritionally balanced MYO options, is recommended to understand the extent to which the provision of different options alters food selection. These examinations should explore the effect of MYO options during lunch, when diners generally have less time to prepare their own meal, and convenient alternatives in the evening. Research in a variety of food service environments (beyond a buffet setting and outside an educational setting), and studies introducing convenient and appealing healthful alternatives amongst varied assortments is recommended. Beyond testing of a broader range of structural changes, the inclusion of labelling on any introduced alternatives should be examined. Additionally, consideration of the impact food service changes may have on social elements represents an opportunity for future research, given that the food environment can directly support socialisation, which in turn can nurture relationships [60,61].

The impact of the new food alternatives provided as part of the structural changes was only assessed via the direct observation method and were not included in the plate photography analysis—representing an opportunity for future research. In other words, including the new alternatives in the determination of healthfulness via the plate photography method is recommended. This could extend to the estimation of the nutrient composition of all foods, rather than the classification scheme reported in this paper and in the paper by Carins et al. [28]. Future assessments need to assess the effect of introducing new options while controlling the variability of existing offerings; as well as estimation of portion size, and food left unconsumed (wastage) to better determine food consumption following structural changes, given that all food selected may not be consumed. Furthermore, increasing the size or representation of the plate photography sample would give more confidence that the sample is representative of the total dining population. Finally, the impact of different environmental changes (e.g., furniture, room colour and other atmospheric aspects) could be tested using an additive design to ascertain which components exert the greatest effect on diner behaviours.

Observation figures indicated that some diners returned to select a second main dish. This was not normal practice and was discouraged by the caterers at both time points, so future research should track individuals to ascertain how often this occurs. Given that payment generated a paper ticket, which was used to visually indicate to the caterer the diner’s entitlement to a meal when dropped into the container (at the beginning of the mains counter; and at the paper bag lunch and MYO counters post changes), some effort was required by any diner engaging in this practice. Modification to the ticketing process may reduce the likelihood of this occurring in future studies. 

Further still, studies that examine diners’ preferences for new alternatives, satisfaction and repeated patronage would be informative. These studies should determine the effect existing preferences have on the selection of new alternatives, and whether the novelty of new alternatives diminishes over time. They should also explore the impact of available time, perceptions of whether the new alternatives represent value for money, and whether sit down or take away options are preferred; especially as these factors may differ between lunch and dinner. Finally, future research should include the examination of the cost of new alternatives, and the impact this has on profits for food providers. Clear case studies that demonstrate how food service structural changes designed to improve consumer health can positively affect the retailer’s bottom line would provide compelling evidence to alleviate concerns held by retailers, thereby encouraging structural change.

## 5. Conclusions

Environmental factors play a significant role in dietary behaviour. The food service sector represents a promising avenue through which dietary behaviours can be improved. However, a holistic understanding is needed to ensure both consumers and retailers receive mutual benefit (not loss of revenue or profit). Observational studies that track food choices, sales of healthy alternatives, consumer satisfaction, repeat/return patronage as well as profit would provide well-rounded justifications for how the food service sector can make positive changes. Such evidence is an important means to engaging the food service sector, and to encourage service structural changes that benefit individuals, communities, businesses and society.

## Figures and Tables

**Table 1:** Food counter usage by the diners, before and after, assessed by direct observation.

**Lunch**	**Food Counter**	**Before (*n* = 1294)**	**After (*n* = 1230)**
		**Usage (%)**	**Usage (%)**
	Paper Bag Lunch	-	230 (19%)
	Main dish	1201 (93%) **	1049 (85%) **
	Hot Vegetable	972 (75%) *	974 (79%) *
	Salad bar	857 (66%) ^ns^	811 (66%) ^ns^
	Sandwich bar	988 (76%)**	458 (37%)**
**Dinner**	**Food Counter**	**Before (*n* = 787** **)**	**After (*n* = 843** **)**
		**Usage (%)**	**Usage (%)**
	MYO Pizza	-	244 (29%)
	Main dish	774 (98%) **	648 (77%) **
	Hot Vegetable	709 (90%) **	556 (66%) **
	Salad bar	463 (59%) **	403 (48%) **
	Sandwich bar ^1^	469 (60%)	-

^1^ The sandwich bar was not made available during the dinner meal after implementation; * Difference between groups (before vs. after) significant at *p* = 0.05 level; ** Difference between groups (before vs. after) significant at *p* = 0.01 level; ^ns^ No significant difference between group means (before vs. after) at *p* = 0.05 level.

**Table 2:** Number of buffet selections, per plate, before and after, assessed by plate photography.

**Lunch**	**Selections** ^1^	**Before (*n* = 159)**			**After (*n* = 224)**		
		**Mean**	**(95% CI)**	**Mode**	**Mean**	**(95% CI)**	**Mode**
	Red	0.65 **	(0.53, 0.77)	0	0.86 **	(0.76, 0.96)	1
	Orange	1.04 **	(0.92, 1.16)	1	1.45 **	(1.32, 1.58)	1
	Green	3.87 **	(3.61, 4.14)	4	2.97 **	(2.75, 3.19)	2
	Total	5.56 ^ns^	(5.27, 5.85)	5	5.27 ^ns^	(5.04, 5.50)	4
**Dinner**	**Selections**	**Before (*n* = 139)**			**After (*n* = 51 ^2^)**		
		**Mean**	**(95% CI)**	**Mode**	**Mean**	**(95% CI)**	**Mode**
	Red	0.40 **	(0.31, 0.50)	0	1.31 **	(1.08, 1.55)	1
	Orange	1.15 **	(1.03, 1.28)	1	0.67 **	(0.49, 0.84)	2
	Green	3.94 **	(3.65, 4.23)	4	3.18 **	(2.80, 3.56)	3
	Total	5.50 ^ns^	(5.21, 5.78)	5	5.16 ^ns^	(4.75, 5.56)	5

^1^ Red=Least healthful; Orange=Moderately healthful; Green=Most healthful; ^2^ Sample size for dinner meal after implementation was greatly reduced due to uptake of the pizza option; ** Difference between group means (before vs. after) significant at *p* = 0.01 level; ^ns^ No significant difference between group means (before vs. after) at *p* = 0.05 level.

**Table 3:** Number of options ^1^ provided in the buffet, by health category, before and after, sourced directly from the buffet menu.

**Lunch**	**Number of Options Provided**	**Before *n* (%)**	**After *n* (%)**
	Red	13 (16%)	14 (17%)
	Orange	21 (26%)	21 (25%)
	Green	47 (58%)	48 (58%)
	Total	81 (100%)	83 (100%)
**Dinner**	**Number of Options Provided**	**Before *n* (%)**	**After *n* (%)**
	Red	10 (14%)	11 (25%)
	Orange	17 (25%)	6 (14%)
	Green	42 (61%)	27 (61%)
	Total	69 (100%)	44 (100%)

^1^ Number of options refers to dishes provided in the buffet, and does not include new meal options.

## Appendix A. Healthfulness Classification of Foods Made Available for Selection

Each dish from the menu was classified using a healthfulness scheme published elsewhere [28]. Every variant was assessed. Some foods can be classified immediately (e.g., plain vegetables are always classified green); others can only be classified after nutrient values are obtained using the most generic recipes for the dish. The tables below summarise the classification outcomes, presenting similar variants together (e.g., all leans meats such as chicken, beef and fish) are grouped together.

**Table A1:** Summary of healthfulness classifications of dishes made available at lunch.

Food Counter	Type of Dish	Rating	Number (before)	Number (after)
Main	Meat (lean/trimmed)	Green	1	0
Main	Meat (non-trimmed) or mixed dishes (casserole/stir fry) below fat/salt limit	Orange	11	8
Main	Deep-fried food or Mixed dishes (casseroles/stir fry) above fat/salt limit	Red	5	7
Vegetable	Plain potato	Green	1	1
Vegetable	Mashed potato	Orange	0	2
Vegetable	Roast potato (added salt/oil/fat)	Orange	0	0
Vegetable	Potato fries or potato bake (added cream or cheese)	Red	1	2
Vegetable	Plain vegetables	Green	8	8
Vegetable	Roast vegetables (added salt/oil/fat)	Orange	0	1
Vegetable	Plain rice	Green	2	1
Vegetable	Fried rice/noodles	Orange	0	2
Salad	Plain salad/vinegar dressed salad or separate salad vegetables	Green	18	19
Salad	Plain fruit pieces	Green	6	10
Salad	Creamy dressed salad (vegetable)	Orange	5	5
Salad	Creamy dressed salad (added processed meat or cheese)	Red	1	0
Sandwich	Bread	Green	6	4
Sandwich	Sliced lean meat	Green	2	3
Sandwich	Hard-boiled egg	Green	2	1
Sandwich	Wrap/sandwich portions ^1^ (vegetable only)	Green	1	1
Sandwich	Egg/tuna mayonnaise mix	Orange	2	2
Sandwich	Cheese	Orange	2	2
Sandwich	Wrap/sandwich portions ^1^ (cheese/egg mayonnaise mixes)	Orange	1	0
Sandwich	Processed meat	Red	5	3
Sandwich	Wrap/sandwich portions ^1^ (processed meat)	Red	1	1

^1^ For some meals, caterers offered premade wrap portions, or small sandwiches. These were not as large as those offered in the paper bag lunch.

**Table A2:** Summary of healthfulness classifications of dishes made available at dinner.

Food Counter	Type of Dish	Rating	Number (before)	Number (after)
Main	Meat (lean/trimmed)	Green	1	0
Main	Meat (non-trimmed) or mixed dishes (casserole/stir fry) below fat/salt limit	Orange	7	2
Main	Deep-fried food or Mixed dishes (casseroles/stir fry) above fat/salt limit	Red	4	7
Vegetable	Plain potato	Green	2	0
Vegetable	Mashed potato	Orange	0	0
Vegetable	Roast potato (added salt/oil/fat)	Orange	1	0
Vegetable	Potato fries or potato bake (added cream or cheese)	Red	0	4
Vegetable	Plain vegetables	Green	8	6
Vegetable	Roast vegetables (added salt/oil/fat)	Orange	1	0
Vegetable	Plain rice	Green	2	1
Vegetable	Fried rice/noodles	Orange	0	0
Salad	Plain salad/vinegar dressed salad or separate salad vegetables	Green	15	10
Salad	Plain fruit pieces	Green	9	7
Salad	Creamy dressed salad (vegetable)	Orange	4	3
Salad	Creamy dressed salad (added processed meat or cheese)	Red	1	0
Sandwich	Bread	Green	3	3^1^
Sandwich	Sliced lean meat	Green	2	0
Sandwich	Hard-boiled egg	Green	0	0
Sandwich	Wrap/sandwich portions (vegetable only)	Green	0	0
Sandwich	Egg/tuna mayonnaise mix	Orange	2	0
Sandwich	Cheese	Orange	2	1^1^
Sandwich	Wrap/sandwich portions (cheese/egg mayonnaise mixes)	Orange	0	0
Sandwich	Processed meat	Red	3	0
Sandwich	Wrap/sandwich portions (processed meat)	Red	2	0

^1^ Bread and sliced cheese were served in the salad counter, at dinner, after changes.
